# Exploring the influence of culture on the physical activity routines of elderly individuals with chronic diseases: a scoping review

**DOI:** 10.1186/s12889-025-24616-w

**Published:** 2025-10-28

**Authors:** Lamya Alrashidi, Gillian Prue, Iseult Wilson

**Affiliations:** 1 School of Nursing and Midwifery, Queen’s University Belfast, Belfast City, United Kingdom; 2 College of Nursing & Midwifery, Mohammed Bin Rashid, University of Medicine and Health Sciences, Dubai City, UAE

**Keywords:** Physical activity, Chronic diseases, Older adults, Culture, And beliefs

## Abstract

**Background:**

The global demographic shift towards a population with a more significant proportion of elderly individuals has been associated with notable advancements in medical technology, leading to an increased incidence of chronic illnesses. The importance of healthcare and regular physical activity in preventing illnesses among older adults was emphasised. Consistent participation in exercise has been shown to provide benefits such as decreased vulnerability, lower mortality risk, and improved cognitive abilities. Even with the advantages, there were challenges linked to maintaining a regular exercise routine. The primary objective of this study was to address the current gap in research about the impact of culture on exercise behaviours in the population of older individuals who suffer from chronic diseases. The concept of culture, as published by UNESCO, was used to carry out a comprehensive investigation.

**Methods:**

The main objective of this study is to investigate the influence of cultural variables on the level of participation in physical exercise among elderly adults with chronic illnesses. The research used the framework proposed by Arksey and O’Malley and will conform to the PRISMA-ScR guidelines for documenting systematic reviews. The study used a rigorous process to identify relevant scientific publications published in either English or Arabic during the last twenty years. The accomplishment was achieved by extensively searching many databases, such as OVID Medline, Embase, CINAHL, and Scopus. The investigation includes the period up to June 2023, supported by a meticulous assessment of reference lists to ensure a complete study of the literature.

**Results:**

The scoping study identified different cultural factors that have a substantial impact on the physical activity habits of older persons with chronic illnesses. Perspectives towards physical activity are influenced by cultural values and beliefs, including ideas of ageing and health. Traditional techniques, such as Tai Chi, are favoured by elder Chinese Americans due to their cultural alignment and ability to increase involvement. Community-based initiatives that include physical activity into everyday schedules, bolstered by local customs and social connections, substantially enhance engagement and compliance. Familial and social networks played a vital role as important motivators, offering both encouragement and practical assistance. In addition, making environmental changes, such as establishing inclusive and culturally suitable communal areas for physical exercise, plays a crucial part in encouraging physical activity. The results underscore the necessity of implementing culturally sensitive, community-oriented, and environmentally supportive approaches to promote physical activity among older persons who have chronic illnesses.

**Conclusion:**

This scoping study emphasises the significant influence of cultural influences on the physical activity habits of older persons with chronic illnesses. Efficient interventions should possess cultural sensitivity, be rooted in the community, and receive support from familial and social networks. Customised programmes that integrate local traditions, include community people, and adapt the surroundings to promote physical activity are crucial. Further investigation is warranted to delve into these cultural aspects to devise more efficient and comprehensive health promotion tactics.

**Supplementary Information:**

The online version contains supplementary material available at 10.1186/s12889-025-24616-w.

## Background

Advances in medical research have extended the average lifespan of people’s lives, resulting in an increasingly older worldwide population [1]. The shift in demographics has resulted in a higher occurrence of chronic illnesses, which has placed substantial economic strain on healthcare systems worldwide. As a result, regular physical exercise has become a crucial component of contemporary medical treatments, with healthcare practitioners integrating physical activity into therapeutic approaches to prevent and control different diseases [2].

Physical activity refers to any movement of the skeletal muscles that requires energy expenditure, encompassing work, play, domestic duties, travel, and recreational activities. Exercise, a subset of physical activity, is defined as planned, structured, and repetitive movements specifically undertaken to improve or maintain physical fitness and health [3]. Regular physical exercise provides older persons with many advantages, such as decreased vulnerability to noncommunicable diseases, reduced chances of death, and enhanced physical and emotional well-being. Additionally, it aids in reducing cognitive decline by improving memory, mental processing speed, and attention [4].

The World Health Organisation (WHO) has released guidelines recommending higher levels of physical activity and less sedentary behaviour to enhance older individuals’ well-being. According to the World Health Organisation (WHO), it is recommended that people engage in 150–300 min of moderate-intensity or 75–150 min of vigorous-intensity aerobic physical activity per week. Alternatively, they can choose to do a combination of both [5]. The American Heart Association and the American College of Sports Medicine recommend that older persons participate in a minimum of 30 min of physical exercise at a moderate intensity on most days of the week to preserve their health and reduce the risk of chronic illnesses [6].

The elderly’s physical exercise behaviours are notably impacted by culture, especially in individuals with chronic conditions. According to UNESCO, culture refers to the unique spiritual, material, intellectual, and emotional characteristics of a civilization or social group, encompassing its lifestyles, values, customs, and beliefs [7]. The commencement and maintenance of physical activity routines can be significantly influenced by cultural norms and societal expectations. For example, cultural views that prioritise age-related frailty might limit the physical activities of older individuals by influencing how they perceive themselves and how the community perceives their capabilities [8].

In contrast, societies that place importance on, and actively support the involvement of older adults in societal tasks, tend to foster a greater level of engagement and physical activity among this age group. Cultural traditions not only improve personal well-being but also boost the overall welfare of the community, underscoring the significance of cultural knowledge in health promotion efforts targeted towards elderly individuals [9].

Research indicates that physical activities reflecting the diverse cultural backgrounds of individuals are crucial [9]. For instance, many older Chinese Americans choose culturally meaningful practices like Tai Chi, and this choice is based on its potential advantages for physical and mental well-being [9]. This approach emphasises the importance of culturally suitable fitness programmes, accommodating older groups’ preferences and beliefs. Gaining insights into cultural dynamics is crucial for formulating effective health interventions that promote physical activity and enhance the overall health and longevity of the ageing population. The scoping review aims to identify the cultural factors that influence participation in physical activity in this population and summarise the available research [9].

## Materials and methods

A scoping review is a methodical approach that entails discovering and assessing prior research on a certain topic, according to established standards for eligibility. The objective of the scoping review is to synthesise, evaluate, and present the results that directly pertain to a specific research inquiry [10]. Conducting this scoping review is supported by the necessity to investigate the intricate and numerous notions of ‘culture’ and ‘physical activity routines’ among older adults with chronic illnesses. Scoping reviews are well-suited for clarifying concepts and surveying current research. This corresponds with our objectives of identifying areas where knowledge is lacking and guiding future research in this relatively unexplored field. This technique will enable us to efficiently address the wide scope of the research subject and offer a preliminary evaluation of the existing literature [10].

This scoping protocol design is based on the five-stage methodological framework that was designed by Arksey and O’Malley (2005) [11]and subsequently enhanced by Levac et al. (2010) [12]. These five stages are: (1) identifying the research question (2), identifying relevant studies (3), study selection (4), charting the data, and (5) collating, summarising, and reporting the results. This study followed the principles outlined in the Preferred Reporting Items for Systematic Reviews and Meta-Analyses extension for Scoping Reviews to guarantee accuracy in reporting the research findings [13]. See Additional File 1 for the PRISMA-ScR checklist.

### Identifying the research questions

Beginning with the definition provided by UNESCO, the main objective of this research was to comprehensively review and synthesise the theoretical literature on the influence of culture on the physical activity routines of older individuals with chronic diseases. The evaluation is structured around one fundamental question:

- What are the cultural determinants that impact engagement in physical activity among this demographic?

### Identifying relevant studies

The population, concepts, and context methods were adopted to establish the search criteria in Table 1. The inclusion criteria were original research studies published in English or Arabic during the last two decades. These articles had to have a quantitative, qualitative, or mixed-methods design and focus on examining culture’s impact on physical activity behaviours in older persons with chronic diseases.

**Table 1:** The PCC model

Acronym and Components	Description of Components
Population (P)	Older adults aged 65 years and older suffering from chronic diseases
Concepts (C)	Examination of cultural factors and their impact on physical activity behaviours
Context (C)	Studies across diverse global cultural contexts regarding physical activity adherence

To develop the search strategy for the scoping review, a review of the terms derived from the research questions was made utilising the PCC (Population, Concepts, and Context) framework. A thorough collection of search phrases was produced by collaborating with a research team and a subject librarian. A comprehensive compilation of keywords and their various combinations was established to direct the exploration of relevant studies, as outlined in Table 2.

**Table 2:** Terms and relevant synonyms

Terms	The Relevant Synonym
Physical Activity	‘Physical Activity’, ‘Exercise’
Chronic Diseases	‘Chronic Diseases’, ‘Chronic Illness’
Older Adults	‘Older Adults’, ‘Geriatric’, ‘Elderly’
Culture	‘Culture’, ‘Beliefs’

A comprehensive search method was utilised to investigate the impact of cultural influences on physical activity among older individuals with chronic illnesses. This approach incorporated the search phrases and their corresponding synonyms from Table 2 by utilising Boolean operators, as outlined in Table 3. The searches were performed on multiple databases, including CINAHL, OVID Medline, Embase, and Scopus, until June 2023. The selection of these databases was based on their comprehensive coverage of medical and health sciences literature. To conduct a comprehensive analysis of the existing research, the search was augmented by manually reviewing the reference lists of pertinent papers. The systematic methodology was designed to encompass a wide range of investigations and conduct a comprehensive analysis of the data.

**Table 3:** Search strategy with boolean and/or

Keywords and synonyms	Search with Boolean AND/OR
Facilitators, Barriers, Factors	‘Facilitators’ OR ‘Barriers’ OR ‘Factors’
Culture, Beliefs	‘Culture’ AND ‘Beliefs’
Physical Activity, Exercise	Physical Activity’ OR ‘Exercise’
Chronic Diseases, Chronic Illness	‘Chronic Diseases’ OR ‘Chronic Illness’
Older Adults, Geriatric, Elderly	‘Older Adults’ OR ‘Geriatric’ OR ‘Elderly’

### Study selection

During the study selection stage of the scoping review, primary research was carefully examined to ensure it met specific inclusion criteria focusing on the overlap between cultural and physical activity in older persons with chronic conditions. Each study was required to investigate the impact of culture, as defined by UNESCO, on physical activity behaviours within this specific population. To maintain the relevance and timeliness of the review, only research published in English or Arabic within the last 20 years was included. Additionally, the inclusion criteria strictly targeted adults aged 65 years and older. Studies that included mixed-age groups were considered only if they were studies that explicitly distinguished findings by age. This approach ensured that all extracted data directly reflected the experiences of individuals aged 65 and older. By adopting this strategic approach, the scoping review was able to compile extensive and high-quality research, providing a deep understanding of how cultural influences shape the health behaviours of older persons with chronic diseases.

### Critical appraisal for included studies

Following the JBI Scoping Review approach, we did not conduct a formal risk of bias assessment, as scoping research focuses on the mapping of existing literature over the assessment of research quality. To guarantee the thoroughness and dependability of our review, we utilised the Critical Appraisal Skills Programme (CASP) checklist as a systematic tool to evaluate methodological transparency. Every study presented was rigorously assessed using CASP criteria to evaluate its methodological rigour [14]. The appraisal examined studies that employed qualitative, quantitative, and mixed-methods approaches. The CASP Qualitative Checklist was utilised for qualitative studies to evaluate various aspects including the clarity of research objectives, suitability of the methodology, research design, recruitment strategy, data collection, researcher-participant relationships, ethical considerations, the rigour of data analysis, clarity of findings, and overall research value [14]. The CASP Cohort Study Checklist was used to assess quantitative studies. This checklist evaluated various elements including study objectives, research methods, study design, recruiting approach, data collecting, potential confounding factors, completeness of follow-up, accuracy of results, and the relevance of the findings [14]. The mixed-methods studies were evaluated using both qualitative and cohort study checklists to ensure a thorough assessment.

The critical evaluation revealed several strengths and flaws throughout the trials. Typical strengths encompass well-defined study objectives, suitable research approaches, and meticulous data analysis. Nevertheless, numerous studies have shown shortcomings, including insufficient information regarding the connections between researchers and participants. These findings offer a framework for understanding the outcomes of this scoping assessment and emphasise areas that need to be enhanced in future research. Detailed results of the critical appraisal for each included study are provided in Additional File 2.

### Charting the data

The data analysis began with the extraction of pertinent data from the chosen publications: first author, year of publication, study location, research aims, study design and methodology, participant characteristics, outcome measures, and main findings that were in line with the objectives of the scoping review. The researchers used the data extraction tool created by Peters et al. (2020) of the Joanna Briggs Institute [15].

### Collating, summarising, and reporting the results

Relevant studies from the initial research were identified and uploaded into EndNote. Duplicate entries were removed, and the articles were classified as ‘relevant’, ‘not relevant’, or ‘potentially relevant’ based on the information provided in their titles and abstracts. A total of 1,503 articles were initially retrieved. After eliminating duplicate entries, the number of unique publications decreased to 1,262. The first author (LA) included a total of 82 publications in the first trawl based on the titles. Following an abstract screening, 42 articles were retained for further analysis. Each of these 42 papers (full-text) was uploaded into Rayyan and reviewed initially by the first author, and subsequently and independently, by one of the other two authors (GP and IW). In this way, all papers were grouped according to their relevance based on the inclusion criteria, and those in the ‘potentially relevant’ group, were discussed within the team until a consensus was reached.

**Fig. 1:**
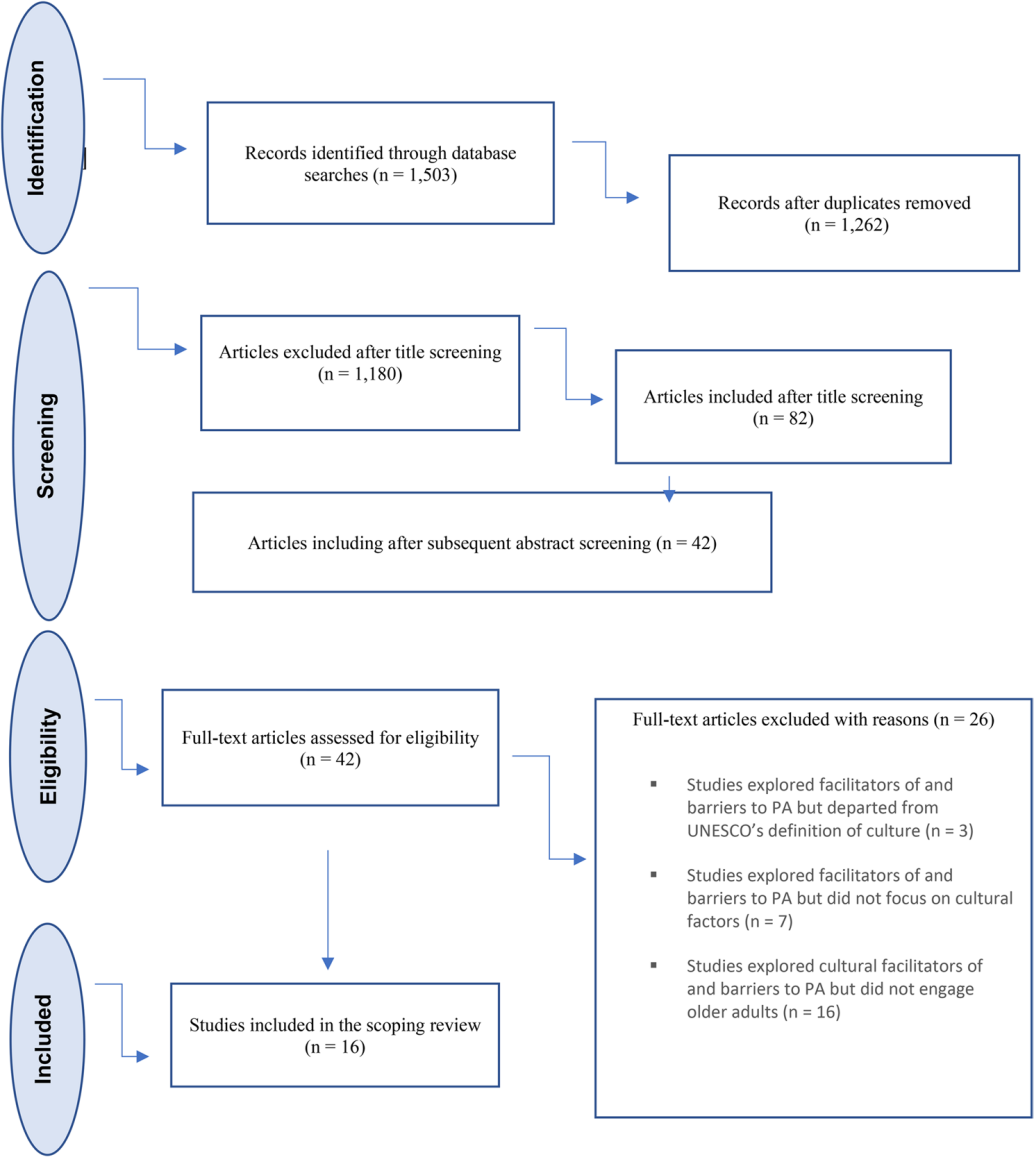
PRISMA flow diagram adapted from [16]

Twenty-six papers did not meet the inclusion criteria, so 16 were included in this review because they met the inclusion criteria. The data were extracted from these 16 articles by (LA). The PRISMA-ScR flow diagram in Fig. 1conveys the outcomes of the literature search and study screening procedure.

## Results

### Description of the studies

A total of 16 studies published between 2004 and 2022 were included in this scoping review (Table 1). The studies were conducted in 10 different countries/regions: Australia (*n* = 2), the United States (*n* = 3), the United Kingdom (*n* = 1), China (*n* = 3), Canada (*n* = 2), Germany (*n* = 1), Japan (*n* = 1), South Asia (*n* = 1), New Zealand (*n* = 1), and Taiwan (*n* = 1). These studies used different research methods: 12 were qualitative, two were quantitative, and 2 were mixed methods. The most common data collection methods were questionnaires (*n* = 4) and individual or focus group interviews (*n* = 14). These data are presented in Table 4. The data were extracted from the studies based on cultural factors that are aligned with UNESCO’s definition in different contexts to identify cultural factors’ diversity. These data are presented in Table 5. In addition, the types of physical activities preferred by older adults are presented in Table 6.

**Table 4:** The main characteristics of the studies

Authors Year Country	Title		Aim	Design	Method	Participants	Outcome measures	Number of participants
Katigbak et al. 2020 US [9]	Older Chinese Americans’ perspectives on physical activity: a mixed methods study		To better understand culturally bound attitudes, behaviours, and beliefs related to PA among older CA. The results could inform design considerations of researchers developing culturally tailored PA interventions for older CA.	Mixed methods	Focus Group & Survey	Older adults	1. The Lubben Social Network Scale 2. The PA Scale for the Elderly, Chinese Version (PASE-C)	60
Belza et al. 2004 US [17]	Older adult perspectives on physical activity and exercise: voices from multiple cultures		To examine barriers to and facilitators of physical activity and exercise among underserved, ethnically diverse older adults.	Qualitative	Focus Group	Older adults		71
You et al. 2021 Australia [24]	Ethnic differences in barriers and enablers to physical activity among older adults		To examine the perceived benefits of, barriers to, and enablers of PA from the perspectives of older Caucasian and Chinese adults living in Australia	Qualitative	1:1 semi-structured interviews	Caucasian and Chinese adults aged 65 or older		64
Schmidt et al. 2016 Canada [8]	Exploring beliefs around physical activity among older adults in rural Canada		To understand the socio-ecological factors that influence or contribute to physical activity among rural-dwelling older adults in Saskatchewan, Canada	Qualitative	1:1 semi-structured interviews	Adults aged 69–94		10
Zhou et al. 2017 China [25]	How the built environment affects change in older people’s physical activity: a mixed- methods approach using longitudinal health survey data in urban China		To examine how individual, social, and physical aspects of the built environment can motivate older people to initiate, regulate, and maintain PA.	Mixed methods	Survey & interviews	Older adults	1. A six-year longitudinal health survey (2010–2015) of older adults 2. Interviews	3,102 (Survey)84 (interviews)
Fuller et al. 2010 Australia	Active living—the perception of older people with chronic conditions		To describe and understand factors that enhance and impede participation in physical activity among older adults with and without chronic illnesses and develop a framework of health behaviours for ‘active living’	Qualitative	Focus Group	Older adults		81: 35 males and 46 females
Clarke et al. 2015 UK [21]	Motivations and barriers to exercise in chronic kidney disease: a qualitative study		To understand CKD patients’ motivators for, barriers to, and beliefs held about exercise	Qualitative	Focus Group	CKD patients		36 aged 26–83
Stehr et al. 2021 Germany	Beliefs and motivation regarding physical activity among older adults in Germany: results of a qualitative study		To gain insights into the belief systems underlying older adults’ physical activity routines and their interplay with motivation by integrating the theory of planned behaviour and self-determination theory.	Qualitative	1:1 semi-structured interviews	Adults aged 65 years or older		20
Song et al. 2019 China	Facilitators and barriers to exercise influenced by traditional Chinese culture: a qualitative study of Chinese patients undergoing haemodialysis		To identify both facilitators of and barriers to exercise among Chinese patients undergoing haemodialysis	Qualitative	1:1 semi-structured interviews & Survey	Chinese patients aged 25–77 years	The Leisure Time Exercise Questionnaire	44
Dave et al. 2013 South Asia	Life stage influences on U.S. South Asian women’s physical activity		To explore SA women’s perspectives on PA during different life stages	Qualitative	1:1 semi-structured interviews	Women aged 18–71 years		42
Yoshigai et al. 2022 Japan	Factors promoting and inhibiting exercise for older women in Kyoto, Japan		To explore promoting and inhibiting factors of exercise among women aged 65 and above in Kyoto city. More specifically, this study aimed to explore the role of possible selves as a facilitator and/or barrier to exercise.	Qualitative	1:1 semi-structured interviews	Japanese women aged 65 and above		42
Chaudhury et al. 2016 Canada	Neighbourhood environment and physical activity in older adults		To examine the relationship of neighbourhood physical and social environment with physical activity among older adults	Quantitative	Survey	Older adults	A cross-sectional telephone survey	434
Purath et al. 2011 US [22]	Physical activity: exploring views of older Russian-speaking Slavic immigrants		To describe culturally embedded motivators of and barriers to physical activity and identify culture- and gender-specific interventions to promote physical activity among older Slavic immigrants	Qualitative	Focus Group	Men and women over 67 years old		4–8
Warbrick et al. 2016 New Zealand [20]	Provider, father, and bro – Sedentary Māori men and their thoughts on physical activity	To understand Māori men’s past and current patterns of physical activity, why they have become sedentary, and what changes they want to make to transition to and maintain a physically active lifestyle		Qualitative	Focus Group	Māori men aged 28–72 years		18
Chen et al. 2015 Taiwan	Identifying factors associated with changes in physical functioning in an older population	To evaluate the association between changes in physical functioning and a variety of factors in an older population in Taiwan		Quantitative	Three-wave cohort study of aging	Older adults		907
Liang et al. 2022 China [18]	Barriers for physical activity in Chinese patients with type 2 diabetes: a qualitative study	To explore contributors to PA deficiency in T2DM patients from the patient’s perspective		Qualitative	Survey & interview	Patients	IPAQ	16

**Table 5:** Cultural aspects that influence engagement in physical activity in different cultural contexts, as defined by unesco’s

Cultural aspects		Spiritual features of society of society	Material features of society	Intellectual features of society	Emotional features of society	Art and literature	Lifestyles and ways of living together	Value system	Traditions	Beliefs
Cultural studies contexts										
Studies in Western contexts										
Australia	• Fuller et al. (2010)						•			
	• You et al. (2021)			•			•			
United States	• Belza et al. (2004)						•			•
	• Purath et al. (2011)						•			•
	• Katigbak et al. (2019)						•	•	•	
United Kingdom	• Clarke et al. (2015)		•				•			
Canada	• Chaudhury et al. (2016)		•				•			
	• Schmidt et al. (2016)						•			
Germany	• Stehr et al. (2021)						•			•
New Zealand	• Warbrick et al. (2016)		•				•			
Studies in Asian contexts										
China	• Zhou et al. (2017)		•				•			
	• Song et al. (2019)						•		•	•
	• Liang et al. (2022)						•			
Japan	• Yoshigai et al. (2022)						•		•	•
Taiwan	• Chen et al. (2015)						•	•		
South Asia	• Dave et al. (2013)	•					•			•

**Table 6:** Types of physical activities preferred by older adults in studies

Studies	Activity type	Description	Cultural significance
Belza et al. (2004) [17]	Walking	Readily available and suitable for all ages.	Wellness and leisure.
Clarke et al. (2015) [21]			
Chaudhury et al. (2016)			
Schmidt et al. (2016) [8]			
Stehr et al. (2021)			
Katigbak et al. (2019)	Traditional Exercises	Includes practices like tai chi, which are deeply connected with Chinese culture.	Enhancing well-being and spiritual balance.
Clarke et al. (2015) [21]	Group activities	Includes exercise classes, dance sessions, and sports that are arranged in communal settings.	Facilitates the development of social cohesiveness and engagement among communities.
Katigbak et al. (2019)			
Stehr et al. (2021)			
Schmidt et al. (2016) [8]	Gardening/Outdoor activities	It involves manual effort and is frequently integrated into everyday schedules, particularly in rural environments.	Offers both physical activity and a feeling of achievement.

### Critical appraisal of included studies

The quality of included studies was assessed using the Critical Appraisal Skills Programme (CASP) tools, tailored for qualitative, quantitative, and mixed-method studies. This appraisal evaluated key aspects such as clarity of research objectives, appropriateness of methodology, recruitment strategies, ethical considerations, and rigour of data analysis. Most studies demonstrated high methodological quality, with clear aims, appropriate designs, and robust data collection methods. However, some studies partially addressed the relationships between researchers and participants or did not report these explicitly (Table 7).

**Table 7:** Summary of quality assessment of included studies

Study (Author, Year)	Methodology	Clear Aim	Appropriate Methodology	Ethical Issues Considered	Rigorous Data Analysis	Overall Quality
Belza et al. (2004) [17]	Qualitative	yes	yes	yes	yes	High
Clarke et al. (2015) [21]	Qualitative	yes	yes	yes	yes	High
Dave et al. (2013)	Qualitative	yes	yes	Partially	yes	High
Fuller et al. (2010)	Qualitative	yes	yes	yes	yes	High
Liang et al. (2021)	Qualitative	yes	yes	Partially	yes	High
Purath et al. (2011) [22]	Qualitative	yes	yes	yes	yes	High
Schmidt et al. (2016) [8]	Qualitative	yes	yes	Partially	yes	High
You et al. (2021) [24]	Qualitative	yes	yes	yes	yes	High
Katigbak et al. (2020) [9]	Mixed Methods	yes	yes	yes	yes	High
Zhou et al. (2017) [25]	Mixed Methods	yes	yes	yes	yes	High

A full breakdown of the critical appraisal results remains available in Additional File 2 for transparency.

### Cultural factors influencing physical activity among older adults

The scoping review encompassed 16 studies that investigated the impact of cultural variables on physical activity in older persons with chronic conditions. The investigations covered various cultural contexts, uncovering multiple values, beliefs, customs, lifestyles, perspectives, and the impact of family and social networks on physical exercise habits.

#### Values and beliefs

Several studies have emphasised the impact of cultural values and beliefs on physical exercise. The study conducted by Belza et al. revealed that older persons from various cultural backgrounds recognised the importance of physical activity for their health. However, they encountered obstacles because of deeply rooted cultural views about ageing and physical effort [17]. Liang et al. found that Chinese patients with type 2 diabetes commonly held the belief that engaging in intense physical activity could have negative effects on their health. This belief reflects a cultural value placed on conserving energy and avoiding excessive exertion [18].

#### Traditions and practices

Traditions and norms were also influential. Song et al. found that traditional Chinese health practices, which place importance on achieving balance and harmony, had an impact on the physical activity routines of Chinese patients receiving hemodialysis treatment [19]. These patients frequently favoured low-impact exercises such as Tai Chi, which is consistent with traditional beliefs regarding gentle and harmonious movement. Warbrick et al. observed that sedentary Māori males perceived physical activity in the context of traditional roles and duties, where physical labour was traditionally a part of their daily routine [20].

#### Lifestyle and social norms

Physical activity is significantly influenced by lifestyles and societal norms. Clarke et al. discovered that social support and group activities had a crucial role in motivating chronic renal disease patients to engage in physical exercise [21]. This finding highlights the importance of communal lifestyle, which is commonly observed in various cultures. Purath et al. emphasised that older persons in community-based settings were more inclined to participate in physical activities when these activities were incorporated into their daily routines and endorsed by community norms [22].

#### Perspectives and perceptions

Perspectives and perceptions regarding health and ageing have a substantial impact on physical activity habits. In a study conducted by Dave et al. [23], it was shown that the physical activity of South Asian women in the United States was influenced by their cultural beliefs about body image and health [23]. These women often placed less importance on physical activity due to their household duties and societal expectations. Similarly, a study conducted by You et al. discovered that variations in ethnic backgrounds affected how older persons perceive ageing and physical abilities, which in turn influenced the specific sorts and level of physical activities they engaged in [24].

#### Influence of the built environment

The constructed surroundings also mirrored cultural influences. Zhou et al. highlighted that in metropolitan areas of China, the layout and availability of community spaces had a significant impact on the physical activity levels of older individuals [25]. Traditional Chinese urban planning, characterised by the presence of communal exercise places, promotes physical activity by integrating cultural values and environmental considerations.

#### Influence of familial and social networks

Family and social connections played a crucial role in influencing physical activity levels. Katigbak et al. discovered that family support had a substantial impact on the participation of older Chinese Americans in physical exercise, serving as a strong catalyst [9]. Family members frequently motivated engagement in physical activity and occasionally partook in the activities, creating a collective endeavour. Warbrick et al. observed that peer support and companionship played a vital role in motivating Māori males to engage in physical activity. The influence of peers and the social dimension of exercise had a crucial role in sustaining regular amounts of physical activity [20].

According to Clarke et al., assistance and backing from family and friends played a crucial role in helping chronic renal disease patients overcome obstacles to engaging in physical exercise [21]. The provision of social support, including services such as transportation to exercise facilities or companionship during walks, played a crucial role in motivating and assisting individuals to participate in regular physical activity.

In short, the scoping study concluded that cultural elements such as values, beliefs, traditions, lifestyles, worldviews, and familial and social networks have a significant impact on the physical activity habits of older persons with chronic conditions. The cultural elements, as delineated by UNESCO, have significant impacts on the way older individuals perceive and participate in physical activities.

### Approaches to enhance physical activity in older individuals

This review covered several trials on culturally sensitive physical activity treatments for older individuals with chronic conditions. These interventions targeted cultural characteristics, beliefs, and practices to promote efficacy and acceptance.

#### Culture-sensitive interventions

Culturally customised fitness programmes for older Chinese Americans were successful [9]. These programmes used Tai Chi and Qigong, which participants were familiar with and fit their cultural penchant for low-impact, meditative activities. Family participation was encouraged to create a supportive environment and make physical activity more social.

#### Community-based programmes

A community-based nursing programme to get older people exercising was detailed by Purath et al. This community-supported programme integrated physical activity into everyday activities. The programme enhanced engagement and adherence by including local community members and using social media to promote accountability and involvement [22].

#### Social support and group activities

One study stressed social support and group activities for chronic renal disease patients. The programme organised group activities and social events for exercise and socialisation. This method worked well in societies that emphasise community activities and where social isolation can make exercise difficult [21].

#### Environmental modifications

The study conducted by Zhou et al. explored how built environment changes can encourage older persons to exercise [25]. Their contribution to urban China included creating accessible and safe exercise facilities like parks and communal exercise rooms. These rooms were culturally acceptable, using traditional aspects and making them appealing to older folks.

#### Family engagement

Several research stressed the importance of family involvement in physical activity. Several studies discovered that family members motivated older persons to exercise. Family-led exercise programmes provided additional support and reinforced cultural norms of family togetherness and reciprocal caring [9, 20].

The review concluded that culturally responsive interventions and programmes enhanced physical activity in older persons with chronic conditions. These methods used cultural values, social support, community involvement, and environmental changes.

## Discussion

The results of this scoping study highlight the important influence of cultural influences on the physical activity habits of older persons with chronic illnesses. The studies demonstrate the impact of cultural values, beliefs, customs, lifestyles, worldviews, and familial and social networks. Programs that are sensitive to culture and centred on the community are essential in increasing physical activity among this particular group.

Interventions that are specifically designed to align with a particular culture have demonstrated notable effectiveness. For example, including traditional practices like Tai Chi and Qigong, together with family involvement, is in line with cultural preferences and promotes a nurturing atmosphere [9]. Cairney et al. assert that to improve effectiveness, health promotion efforts should incorporate cultural practices. Cultural sensitivity is important as it not only shows respect for the participants’ origins but also enhances their involvement and commitment to physical activity programmes [26].

Community-based programmes are crucial in promoting physical exercise among older individuals., Purath et al. outlined a programme that successfully incorporated physical activity into everyday schedules, with the assistance of community values and social networks [22]. The strategy of promoting communal participation and accountability led to a substantial rise in engagement and adherence. The effectiveness of such programmes underscores the significance of utilising pre-existing community frameworks to encourage health behaviours. They highlight that social support systems are vital for overcoming obstacles to physical activity [27].

Familial and social networks play a crucial role in shaping physical activity. Many studies highlight the significance of social support and peer networks in motivating elderly individuals to participate in physical activities [20, 21]. Family and peers offer both moral support and tangible aid, such as transportation and companionship, throughout various activities. The social ties have a crucial role in sustaining regular physical exercise among older persons. This underscores the importance of interventions that incorporate both family and peer elements [27].

Environmental alterations are another crucial element, and communal places that are accessible and culturally suitable for physical activity need to be established [25]. These alterations are crucial in metropolitan areas because the constructed surroundings can either enable or impede physical activity. This is consistent with the consensus that the environment has a substantial impact on encouraging healthy behaviours and should be intentionally created to facilitate physical activity that is customised to cultural and community requirements [27]​.

## Strengths and limitations

This scoping review provides an integrative analysis of cultural factors affecting physical activity in older adults with chronic conditions, synthesising findings from various cultural contexts. A significant strength lies in the systematic application of established scoping review methodologies, such as the Arksey and O’Malley framework and PRISMA-ScR guidelines, guaranteeing a transparent and thorough approach to data collection and synthesis. The incorporation of studies across various global regions improves the generalisability of the results.

The study presents certain limitations. First, limiting the language to English and Arabic might have limited the range of cultural viewpoints that were recorded by excluding pertinent research written in other languages. Second, variations in study designs and methodologies created inconsistencies that hindered direct comparisons among studies. Finally, the exclusion of studies that did not disaggregate findings by age may have resulted in the neglect of potentially relevant insights from mixed-age cohorts. Qualitative studies that specifically differentiated findings by age were incorporated to alleviate this limitation as effectively as possible.

## Implication of the findings

The findings from this review highlight the necessity for culturally customised health promotion techniques to effectively promote physical activity among older persons suffering from chronic conditions. Interventions should incorporate conventional physical activities while honouring cultural norms, utilise strategies that involve the local community to encourage involvement and engage family and social support networks to offer encouragement and aid. Furthermore, it is crucial to provide community spaces that are accessible and tailored to specific cultural needs and preferences. Further investigation is warranted to delve into these cultural aspects to devise more efficient health promotion tactics.

## Conclusion

The scoping review highlights the substantial influence of cultural influences on the physical activity behaviours of older persons with chronic conditions. For increasing physical activity in this population, it is crucial to implement culturally appropriate treatments and community-based programmes, provide familial and social support, and make environmental adjustments. Nevertheless, it is vital to confront the obstacles and guarantee that interventions are flexible enough to meet the varied requirements of older individuals. Subsequent investigations should persist in examining and integrating these cultural aspects to optimise the efficacy of health promotion techniques.

## Supplementary Information

Supplementary Material 1.

Supplementary Material 2.

## Data Availability

No datasets were generated or analysed during the current study.
