# Supplementary material for: Exploring the influence of culture on the physical activity routines of elderly individuals with chronic diseases: a scoping review
Source: BMC Public Health. 2025 Oct 28;25:3624. doi: 10.1186/s12889-025-24616-w (PMC12560489; doi:10.1186/s12889-025-24616-w)
Supplement: Supplementary file 2 — Supplementary Material 2. [file 12889_2025_24616_MOESM2_ESM.docx]

**Additional File 2**

**Critical appraisal for included studies using (CASP Tool)**

1. **CASP Qualitative Studies Checklist result in Qualitative studies**

| **Study (Author, Year)** | **Clear Aim** | **Appropriate Methodology** | **Appropriate Research Design** | **Appropriate Recruitment Strategy** | **Data collection addressed Research Issue** | **Relationship Considered** | **Ethical Issues Considered** | **Rigorous Data Analysis** | **Clear Statement of Findings** | **Value of Research** |
| --- | --- | --- | --- | --- | --- | --- | --- | --- | --- | --- |
| (**Belza et al., 2004)** | Yes | Yes | Yes | Yes | Yes | Yes | Yes | Yes | Yes | High |
| **(Clarke et al., 2015)** | Yes | Yes | Yes | Yes | Yes | NO | Yes | Yes | Yes | High |
| **(Dave et al., 2013)** | Yes | Yes | Yes | Yes | Yes | Partially | Yes | Yes | Yes | High |
| **(Fuller et al., 2010)** | Yes | Yes | Yes | Yes | Yes | Yes | Yes | Yes | Yes | High |
| **(Liang et al., 2021)** | Yes | Yes | Yes | Yes | Yes | Partially | Yes | Yes | Yes | High |
| **(Purath et al., 2011)** | Yes | Yes | Yes | Yes | Yes | Yes | Yes | Yes | Yes | High |
| **(Schmidt et al., 2016)** | Yes | Yes | Yes | Yes | Yes | Partially | Yes | Yes | Yes | High |
| **(You et al., 2021)** | Yes | Yes | Yes | Yes | Yes | Yes | Yes | Yes | Yes | High |
| **(Warbrick et al., 2016)** | Yes | Yes | Yes | Yes | Yes | Yes | Yes | Yes | Yes | High |
| **(Yoshigai et al., 2022)** | Yes | Yes | Yes | Yes | Yes | Partially | Yes | Yes | Yes | High |
| **(Song et al., 2019)** | Yes | Yes | Yes | Yes | Yes | Partially | Yes | Yes | Yes | High |
| **(Stehr et al., 2021)** | Yes | Yes | Yes | Yes | Yes | Yes | Yes | Yes | Yes | High |

1. **CASP Cohort studies checklists result in Quantitative studies**

| **Study (Author, Year)** | **Clear Aim** | **Appropriate Methodology** | **Appropriate Research Design** | **Appropriate Recruitment Strategy** | **Data collection addressed Research Issue** | **Relationship Considered** | **Ethical Issues Considered** | **Rigorous Data Analysis** | **Clear Statement of Findings** | **Value of Research** |
| --- | --- | --- | --- | --- | --- | --- | --- | --- | --- | --- |
| **(Chaudhury et al., 2016)** | Yes | Yes | Yes | Yes | Yes | No | Yes | Yes | Yes | High |
| **(Chen et al., 2015)** | Yes | Yes | Yes | Yes | Yes | No | Yes | Yes | Yes | High |

1. **CASP Cohort and Qualitative studies checklists result in Mixed-Methods studies**

| **Study (Author, Year)** | **Clear Aim** | **Appropriate Methodology** | **Appropriate Research Design** | **Appropriate Recruitment Strategy** | **Data collection addressed Research Issue** | **Relationship Considered** | **Ethical Issues Considered** | **Rigorous Data Analysis** | **Clear Statement of Findings** | **Value of Research** |
| --- | --- | --- | --- | --- | --- | --- | --- | --- | --- | --- |
| **(Katigbak et al., 2020)** | Yes | Yes | Yes | Yes | Yes | Yes | Yes | Yes | Yes | High |
| **(Zhou et al., 2017)** | Yes | Yes | Yes | Yes | Yes | Yes | Yes | Yes | Yes | High |
